# The effect of sleep–wake behaviors on the onset of mania in youth: A computational model

**DOI:** 10.1192/j.eurpsy.2026.10180

**Published:** 2026-03-09

**Authors:** Kirill Glavatskiy, Ian B. Hickie, Jacob J. Crouse, William Capon, Ante Prodan, Jan Scott, Kathleen Merikangas, Joanne S. Carpenter, Mathew Varidel, Elizabeth M. Scott, Frank Iorfino

**Affiliations:** 1 Brain and Mind Centre, The University of Sydney, Australia; 2 Institute of Neuroscience, Newcastle University, UK; 3 NIMH Intramural Research Program, National Institute of Mental Health (NIMH), USA

**Keywords:** Agent-based modeling, circadian rhythms, complex systems, bipolar disorder, sleep

## Abstract

**Background:**

Bipolar disorder is a recurrent and disabling condition, with a critical clinical need to prevent transitions from euthymia or depression (normal or low activation states) to mania (a high activation state). This study investigates how disruptions in sleep–wake and circadian rhythms may trigger these high activation states, to inform more effective relapse prevention strategies.

**Methods:**

We developed a computational agent-based model integrating empirical evidence, clinical expertise, and lived experience to simulate how 24-hour sleep–wake behaviors (SWBs) influence manic episodes. Individual characteristics were drawn from the Brain and Mind Youth Cohort (*N* = 2,330), and multiple scenarios were simulated to assess how SWB dynamics affect the emergence and course of mania.

**Results:**

In the absence of all irregularities, no individuals experienced a manic episode. Removing behavioral feedback loops resulted in a substantial reduction in manic episodes and delayed onset. In contrast, eliminating light–dark entrainment slightly increased the frequency of manic episodes, suggesting that seasonal adaptation plays a stabilizing role. When examining components of SWB separately, removing sleep irregularities alone had only a modest effect on mania rates, whereas reducing activity irregularities led to the largest benefit: a significant drop in mean manic episodes, a delay in onset, and preventing mania in 65% of the simulated agent population.

**Conclusions:**

Our findings highlight the value of computational modeling for uncovering causal dynamics in mental health. These specific findings demonstrate how daily irregularities in sleep–wake behavior may be a necessary condition for mania. Targeting behavioral regularity may offer a powerful pathway for prevention and early intervention.

## Introduction

Mental disorders defy simplistic explanatory models. These conditions are complex phenomena that emerge from interactions across multiple domains (e.g., biological, psychological, behavioral, and social) [1–4]. This complexity necessitates a more integrative and holistic framework that can adequately address nuanced, interconnected processes to improve our understanding and capacity for early intervention and prevention. Computational modeling offers the mathematical and computational language needed to develop and test explicitly specified theories [5–7]. This has led to the wider application of network [8–10] and data science approaches [11–13] to understand the nature and course of mental disorders.

Agent-based modeling (ABM) is a computational modeling approach that simulates the interactions of individual entities, called “agents,” within a defined environment to study how complex phenomena emerge from these micro-level components or behaviors [14–16]. In contrast to statistical approaches, which are inductive in nature (inferring the rules from the observed data), the ABM approach is deductive (simulating data from imposed rules). ABMs start with mechanistic rules of interactions between different components of a complex system and their temporal dynamics [17–19]. In this way, an ABM explores how the interaction between a system’s components can lead to the observed behavior of the entire system [20]. Such behavior is often “emergent,” as it cannot be fully explained by the sum of the individual parts or components but instead arises from the interactions and relationships among the components of the system.

In this work, we apply the ABM approach to a complex phenomenon in psychiatry, namely the switch in activation states in bipolar disorder (BD). BD is a serious mental illness that is highly disabling and too often leads to premature mortality [21]. Despite this burden, the cause of BD remains unknown and prevents us from providing effective and timely treatments [22]. While the core pathophysiology of BD remains a matter of debate, there is accumulating evidence in support of a primary role of changes in the patterns of objective motor activation, being a cardinal feature of both mania and depression [23–28]. Changes in 24-hour rhythms of rest, activity, and sleep (called sleep–wake behaviors [SWBs]), are linked to motor activation and are strongly influenced by the circadian (body clock) system. Disturbances in circadian rhythms have been proposed as one factor underpinning the switching between different manic, depressive, and euthymic states [29, 30]. However, the extent to which shifts between activation states are driven solely by circadian dysrhythmias [28] or by other factors [2, 23] (i.e., changes in mood and energy) is a matter of debate.

The important advantage of the ABM approach is that it can be constructed in the absence of complete knowledge about the actual system. As a computational model [31], an ABM explores any possible mechanism of the system’s operation, analyzing the simulated output and selecting those mechanisms which produce realistic outcomes [32–35]. These mechanisms may be grounded in established facts (e.g., people sleep 4–9 hours a day) or in hypothetical assumptions about causal relationships (e.g., less sleep leads to either more or less intensive activity during the day). As a complex systems approach [36, 37], ABMs facilitate the examination of (i) structural nonlinearity between causes and outcomes (e.g., switches between high and low activation states in BD); (ii) presence of feedback loops (causal effects are magnified or dampened as the disorder progresses), (iii) contextual effects (how a disorder is shaped by specific biological and/or environmental factors, e.g., seasonal variations in onset of bipolar disorder); and (iv) its sensitivity to initial conditions (slight differences in biological and environmental conditions may lead to major differences in pathophysiology and course).

Here, we test the hypothesized impacts of SWB on the onset of mania using an ABM framework. To do this, we operationalized the main elements of SWBs and defined criteria for mania, with an overt focus on increased motor activity within 24-hour epochs as the definitional core of the syndrome. This approach does not seek to infer causality from empirical data; rather, it makes causal assumptions explicit by specifying hypothesized mechanisms and examining their consequences within a simulated system. It is important to emphasize, however, that this work is not an exhaustive test of all possible mechanisms for the onset or course of mania. We do not intend to provide a comprehensive account of all elements of individual behavior that may influence mania (such as sleep disturbances or the timing of physical activity) or contribute to mania (such as distractedness or inflated self-esteem). Instead, we focus on a stylized notion of specific SWB mechanisms and mania for initial validation of this approach, with the goal to expand to other mechanistic theories (e.g., mood and cognitive dysfunction) in future work.

## Methods

### Agent population

The available Brain and Mind Centre Youth Cohort consisted of 2,470 people presenting for youth mental health care, of whom 452 were diagnosed with some form of bipolar disorder, of whom 75 were diagnosed with full threshold BD-I, and of whom 22 developed first-onset mania after the initial visit. Among those with BD-I, the mean age at first visit was 19 years (SD = 3.8), 40% were female, and 10% had self-reported family history of BD [38].

Our agent population consists of 200 digital agents (i.e., artificial “individuals”). The agents were initialized based on relevant characteristics derived from empirical data collected from the Brain and Mind Youth Cohort [38]. These were sociodemographic (age and sex), clinical (family history of BD and childhood disorders), and circadian (chronotype and seasonal sensitivity) characteristics (see supplement for details).

### Model overview

The simulation progresses in time in discrete steps, with the agents’ state being updated every step. The step size is 1 hour, and the duration of the simulation is 1 year. The agents in the model are heterogeneous; each agent has a unique set of values of individual characteristics (Supplementary Figure S1 and Supplementary Table S2). These characteristics affect the agent’s instantaneous state and SWB according to the set of rules and environment that are the same for all agents. The resulting simulated actigraphy is unique for each agent.

### Operationalization of sleep–wake behaviors (SWBs)

**Figure 1.**
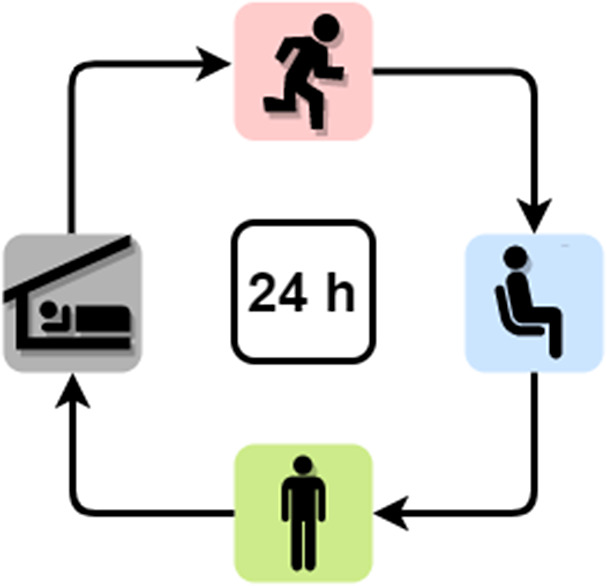
*Sleep–wake behavior (SWB) states.* Starting from the left in the clockwise order: Asleep – nighttime sleep; Active – moderate-to-vigorous physical activity; Sedentary – sedentary behavior; Recess – all other activities.

The SWB of an agent consists of four mutually exclusive *states*, which are experienced for a certain amount of time without interruptions and occur once a day in the listed order: *asleep*, *active*, *sedentary*, and *other* (Figure 1) (Box 1). The agents’ SWB is defined by the timing of each state and is represented by four independent variables: *sleep offset*, *sleep duration*, *activity duration*, and *sedentary duration.* The states are stylized aggregates for various actual behaviors, such as traveling to/from work, exercise, and social interactions. The states and their operationalization align with those from typical sleep–wake studies [39–44] that distinguish the periods of sleep, moderate and vigorous physical activity, and being sedentary (see Supplement for details). These variables quantify SWB and allow unique quantification of the current state at any point in time. The agents’ SWB consists of several components: heterogeneity (each agent has its own unique SWB), behavioral feedback (daily SWB is affected by SWB on the previous day), light–dark entrainment (SWB is affected by the day–night cycle of light), and stochasticity (daily SWB is affected by various random and unpredictable, i.e., stochastic, factors). The SWB model is informed by sleep and activity data from different populations, including healthy and adult cohorts.

Box 1. Terminology.

*Sleep–wake behaviors* (SWBs) – a combination of daytime and nighttime behaviors. These are operationalized as: sleep offset (waking up moment), time of sunrise, and three durations (accumulated over a single day cycle), of night sleep, and intense activities or sedentary periods during the day.

*Sleep behaviors –* one of the following two elements of SWB: sleep duration (i.e., the period of nighttime sleep between sleep onset and sleep offset), and sleep offset.

*Activity behaviors, Activity, Motor activity, Daytime activity* – one of the following two elements of SWB: intense activity (including dedicated physical exercises) and sedentary periods (including doing an office desk job).

*Activation state* – a prolonged pattern of SWB; we identify three such patterns: no mania (typical activation), mania episode (high activation), and remission (typical activation following a period of high activation).

### Operationalization of activation in bipolar disorder

**Figure 2.**
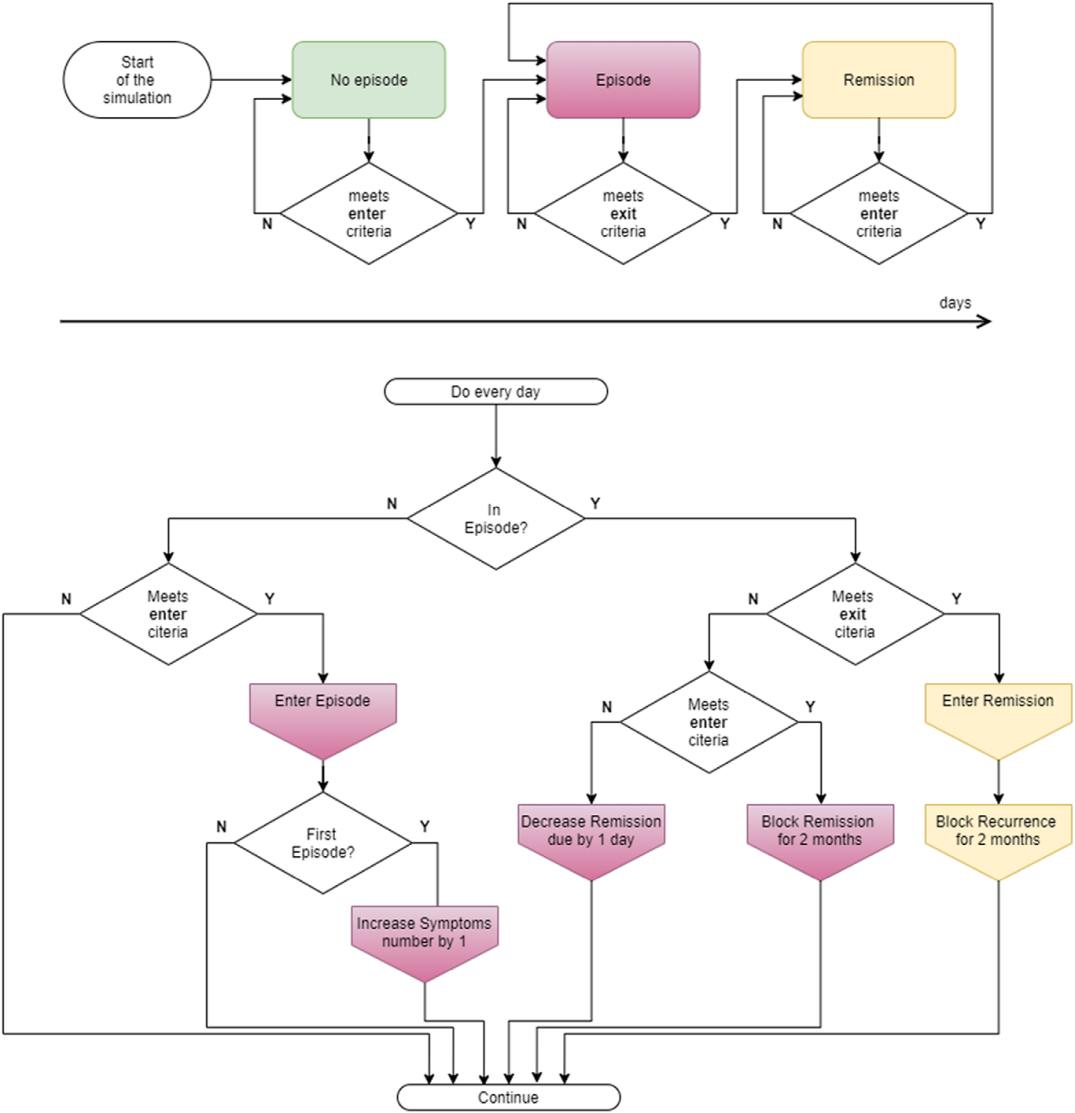
Activation flow chart. Top panel: schematic timeline of different BD states. Bottom panel: Decision process of transitioning between BD states, which happens every day of the simulation. Specifically, each morning, the model calculates the number of symptoms based on the previous day’s behaviors according to Table 1 and compares it to the corresponding threshold values. The model follows a binary decision process, identifying whether an agent is currently in an episode, in which day of the episode/recovery, and so forth, and changes the state of the agent accordingly.

For mania, we identify three activation states of any agent: no mania episode, episode of mania, and remission (Figure 2). The model continuously monitors (by counting symptoms) whether each agent meets the criteria for transition between these states (Figure 2). SWB symptoms are operationalized using the observable DSM-5 criteria for a manic episode (e.g., increase in goal-directed activity and energy, psychomotor agitation, decreased need for sleep, etc.). In this simulation, we assume mood changes compatible with DSM-5 criterion A (Table 1).

**Table 1.** Operationalization of mania activation states and symptoms used in the simulation model

Rules for	Empirical criteria	Operationalization
Episode start (Symptoms of manic episode)	A. A distinct period of abnormally and persistently elevated, expansive, or irritable mood and abnormally and persistently increased goal-directed activity or energy, lasting at least 1 week and present most of the day, nearly every day. B. During the period of mood disturbance and increased energy or activity, three (or more) of the following symptoms are present to a significant degree and represent a noticeable change from usual behavior: 1. Inflated self-esteem or grandiosity. 2. Decreased need for sleep. 3. More talkative than usual or pressure to keep talking. 4. Flight of ideas or subjective experience that thoughts are racing. 5. Distractibility, as reported or observed. 6. Increase in goal-directed activity or psychomotor agitation. 7. Excessive involvement in activities that have a high potential for painful consequences. C and D criteria are not used.	Meets the A criteria. Change in cognition is also present. In addition, more than two of the following are present over a period of at least three consecutive days: The value of sleep duration each day is at most 6 hours. Decrease of sleep duration from day-to-day by at least 0.5 hours. Oscillation (magnitude of decrease or increase) of sleep duration from day-to-day is at least 2 hours. Oscillation (magnitude of decrease or increase) of sleep onset from day-to-day is at least 2 hours. Increase of activity duration from day-to-day by at least 0.25 hours. Presence of an episode in the past.
Episode end	Remission (end of an episode) episode is defined as at least 8 consecutive weeks either with no symptoms of major depression, minor depression, mania, and hypomania, or with only 1 or 2 symptoms of a mild degree and no impairment of psychosocial functioning.	Observing eight consecutive weeks with fewer than three symptoms.
Inter-Episode Period	A new episode cannot start earlier than two months after the previous episode has ended. After the first simulated episode, the risk of a new episode is increased (compared to individuals with no prior manic episode).	Accounted for by an explicit condition in the algorithm.

Each agent starts with one observable symptom corresponding to mood and cognition change, reflecting the studied cohort being initially at risk. Within some period (which is probabilistic and unique for each agent), agents may accumulate the necessary number of symptoms that corresponds to an episode of mania. After the episode finishes, the agent enters remission with increased risk of developing a new episode, which the agent may experience again. The categorical activation state of every agent is updated every day (i.e., no mania, manic episode, or in remission), based on the pattern of symptoms observed over several preceding days (Table 1).

### Model structure

We simulate the dynamics of individual SWB for each digital agent, which is informed by their individual characteristics and common environmental factors. Different scenarios are employed to investigate the impact of these dynamics on the onset and course of mania. While the scenarios are hypothetical, they are designed to reflect relevant behavioral or physiological processes. The goal of the present work is to test mechanistically distinct conditions within an agent-based modeling framework. Accordingly, in each scenario, the entire simulated agent population is exposed to the same scenario assumptions, allowing us to isolate and compare the downstream consequences of specific hypothesized mechanisms under controlled conditions. This means that all model rules, parameters, and scenario conditions were specified a priori. Parameters were calibrated only to establish face validity in a baseline scenario and were held fixed across all subsequent simulations and only modified according to the changing conditions of the scenarios listed below. Schematic representations of each scenario are shown in Figure 4.

Scenario 1: *Baseline (“free-living”*). Reflects the typical SWB for each individual that is expected under normal (i.e., real-world) conditions. This is parameterized using the available scientific literature and actigraphy data [39–44].

Scenario 2: *No light–dark entrainment.* The SWB of each agent is decoupled from the day–night cycle (and the associated changes in the light–dark cycle due to the natural photoperiod), by setting an agent’s seasonal or light sensitivity to zero. This scenario may reflect a person whose circadian system is totally insensitive to the alternating light pattern of the day–night cycle that changes across the year, with longer daylight exposure in summer and shorter daylight exposure in winter.

Scenario 3: *No behavioral feedback.* All temporal associations (i.e., causal links) between SWB variables are set to zero, so that the agent’s behavior on every new day is completely independent of their behavior on the previous day. This scenario represents a person with a free-running endogenous timekeeper that resets every 24 hours, irrespective of external factors.

Scenario 4: *No sleep irregularities.* The sleep part (sleep duration and sleep offset) of the agent’s SWB has no stochastic component. This scenario reflects potential interventions targeted at sleep behavior (e.g., settings with a strict routine of getting out of bed at the same time each day).

Scenario 5: *No activity irregularities.* The activity part (durations of moderate to vigorous and sedentary activities) of the agent’s SWB has no stochastic component. This scenario reflects potential interventions targeted at the activity routine (e.g., behavioral therapy and physical activity interventions).

Scenario 6: *No sleep and activity irregularities.* The SWB of each agent has no stochastic component and is completely deterministic. It is still unique for each agent and may still vary from day-to-day. This scenario may reflect a person who is intrinsically “systematic” (e.g., extremely disciplined) or lives in a strict “systematic” environment (e.g., job/study/sport) that imposes a strict daily routine.

### Model outputs

The outputs of interest for each simulated scenario include the proportion of young people experiencing the first onset of a manic episode, recurrent episodes, and the remaining proportion who do not experience mania. The timing of the episodes is partially framed by the model operationalization and is not assessed in the analysis. In the analysis, we discard the first 10 days of the simulation output to exclude irregularities due to random initialization. We calculate the evolution trajectory for each agent individually, aggregating the results afterwards. The aggregated results are reported in the form of agent population averages and cumulatives, depending on the quantity of interest.

## Results

### Simulation results

**Figure 3.**
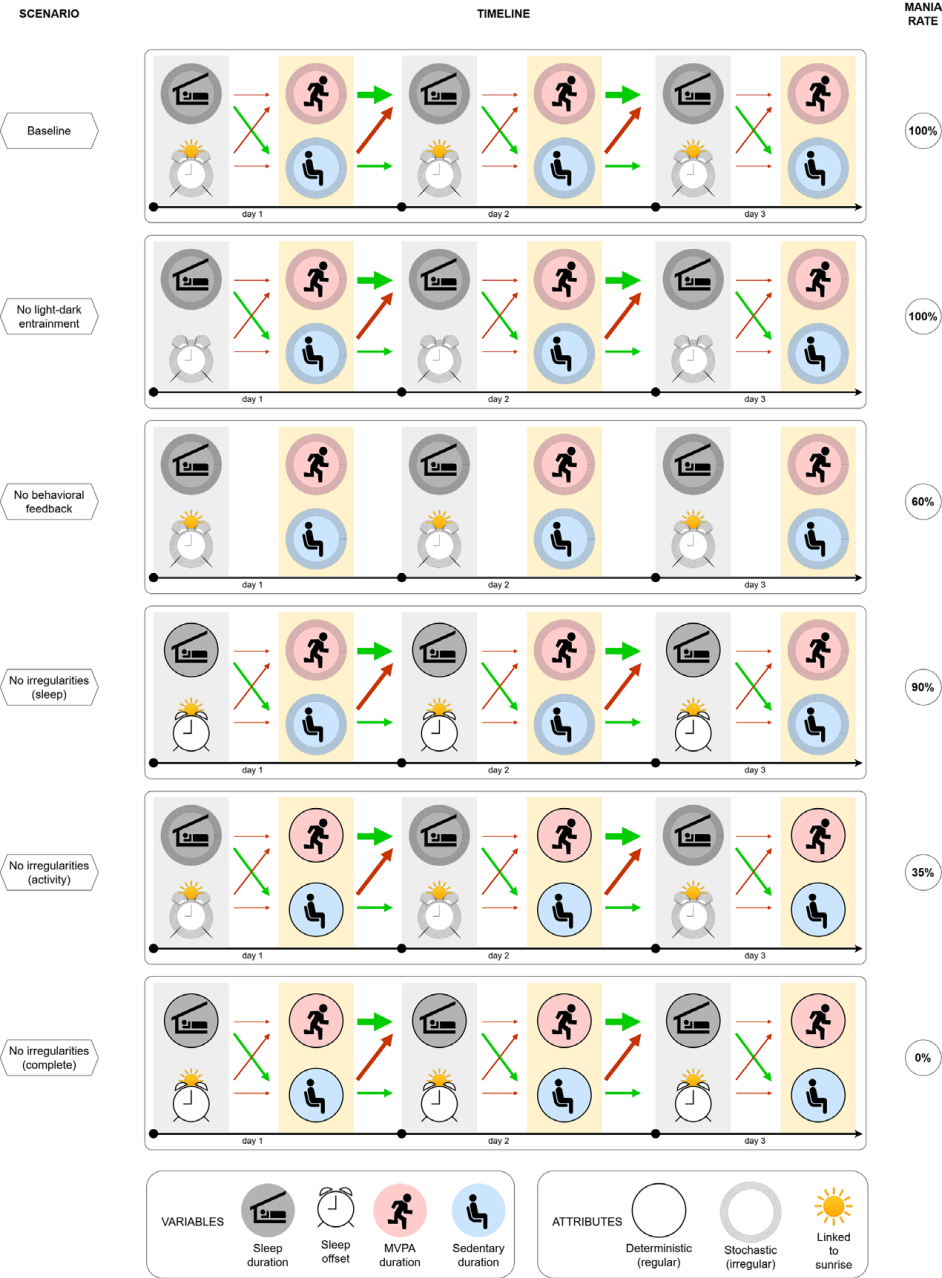
Schematic representation of simulation scenarios. Six panels correspond to the scenarios described in the main text: Baseline, No light–dark entrainment, No behavioral feedback, No sleep irregularities, No activity irregularities, and No sleep and activity irregularities. Each scenario panel illustrates the timeline of the agent’s SWB within and between the days, during the entire simulation, and then reports the probability of having a manic episode over the 12-month period. In any scenario, SWB is represented by the four variables (bottom left panel) that possess certain attributes (bottom right panel) and are related by causal relationships, indicated by arrows (green – positive, red – negative, thickness corresponds to the strength). The baseline scenario contains the “reference” combination of attributes and relationships (as all agents develop mania during the observed 12-month period), while any other scenario differs from the baseline by changing (removing) a single component of the model.

In Scenario 1 (*Baseline*), the typical SWB consisted of 8.09 (SD = 0.41) hours of sleep, 2.06 (SD = 0.10) hours of active behavior, and 9.14 (SD = 0.57) hours of sedentary behavior. Consistent with the clinical cohort on whom the digital agents are modeled, over the course of a year, 100% of agents experienced a manic episode, with a mean of 2.45 (SD = 0.68) episodes in that time frame. The maximum number of episodes within a year was three, with the mean time between the first and the third being 334 (SD = 38) days. The survival curves for the number of episodes in the baseline scenario show self-similarity, with slightly steeper slope after the first episode, indicating that the agents are more vulnerable to subsequent episodes once they have experienced a first full-threshold episode (Figure 3).

**Figure 4.**
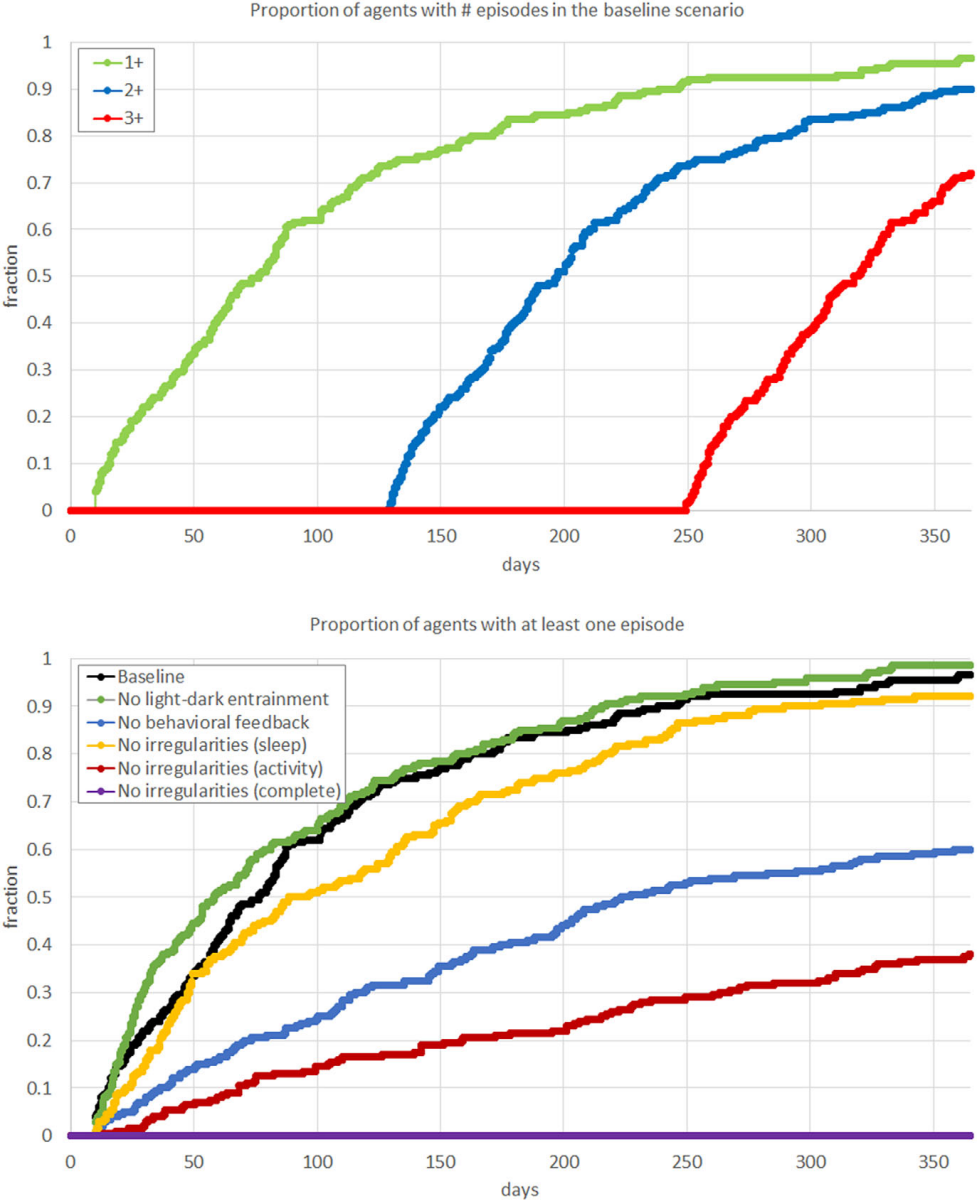
Results of the simulation. Top panel: survival rates for experiencing different numbers of episodes in the baseline scenario, illustrating the reference dynamics of mania progression. Bottom panel: survival rates for experiencing at least one episode in different scenarios described in the main text, illustrating the impact of the interventions in each scenario. The value of the curve at the end of the 12-month period corresponds to the rate reported in the last column of Figure 2. Compared to the baseline scenario, the mania rate is reduced to 60% in the scenario with no behavioral feedback, to 35% in the scenario with no sleep irregularities, and to 0% in the scenario with the absence of any irregularities.

For each scenario, we calculate the cumulative fraction of agents that experienced at least one manic episode within a year (Figure 4).

Substantial variation in daily behavior is observed in scenarios with stochasticity present (Scenarios 1–5). In the absence of any stochastic elements (Scenario 6, *No sleep and activity irregularities*), agents do not experience any manic episode. This suggests that mania cannot develop in a completely controlled environment, with no irregularities in SWB.

In Scenario 2 (*No light-dark entrainment*), we observe a slight increase in the mean number of manic episodes (2.55), suggesting that *appropriate* responsiveness to changes in the light-dark cycle (as happens through changing seasons) reduces the risk of mania.

In Scenario 3 (*No behavioral feedback*), there is a substantial reduction in the mean number of manic episodes (mean = 1.45), and a delay in the time of the first episode (by 28 days), resulting in 42% of the simulated agent population not having an episode of mania. Yet, the variability of the number of episodes increases (SD = 1.362 vs. 0.675 in the baseline scenario). This suggests that SWB causal relationships amplify behavioral irregularities (with prior daily patterns strongly driving subsequent behaviors), thus increasing the number of symptoms and the risk of developing a manic episode.

We further consider scenarios that explore the impact of differential sleep and activity behaviors separately. In Scenario 4 (*No sleep irregularities*), the mania rates show slight improvement compared to the baseline (manic episodes: mean = 2.42, SD = 0.89, 17 days to the first episode, 6% mania-free after 1 year). By contrast, in Scenario 5 (*No activity irregularities*), we observed a significant reduction in the mania rate (mean = 0.68, SD = 1.05), a delay in the timing of the first episode (64 days), and a reduction in the proportion of agents being mania-free after 1 year (65%). We also explored further scenarios where we fixed irregularities of individual variables (i.e., “partial” Scenario 4 and 5), which resulted in the corresponding partial outcomes in mania rates. This suggests that fixing only sleep behaviors does not reduce the risk of mania, and that interventions must address daytime activity behaviors as the key target.

## Discussion

Using a computational model based on simulated data, we show how the onset and course of mania could emerge from disturbances in individual Sleep-Wake Behaviors (SWBs). We selected the type of disruptions that may be important for mania development and operationalized their role in episode onset. No episodes of mania were observed in the absence of daily SWB irregularities in our model. This is consistent with the notion that mania can be associated with changes in behavior (in general) rather than just the initiation of a specific pattern of behavior.

Most importantly, reductions in day-to-day variability (irregularity) in SWB reduce the risk of mania onset or recurrence. Next, enhanced entrainment to the light-dark cycle acts as a stabilizing factor for SWB and hence reduces the risk of mania. By contrast, behavioral (day-to-day and within-day) feedback, acting as a causal system, amplifies prior behavioral disturbances (via positive feedback loops and in the absence of negative feedback loops, which could be preventative) and, hence, increases the risk of mania. That is, once motor activity increases in one 24-hour period, it increases the risk that the behavior is further extended (or amplified) in the following 24-hour period.

Studies have suggested a key role for changes in motor activity among young people with recent-onset BD-I [45], and that alterations in motor activity in bipolar disorders may be less about changes in mean levels of activity and more about changes in day-to-day characteristics of activity [2]. Our findings concur with the hypothesis that dysregulation of the circadian system’s control over the patterns of rest and activity may be a core component of the pathophysiology of mania. Specifically, our findings underscore that the significant influence of regularizing rest-activity rhythms markedly lowers the likelihood of transitioning to mania. Furthermore, we demonstrate the profound impact of positive feedback loops amplifying disturbances. Together, this supports the role of external regulatory systems, such as structured social rhythms and daily routine interventions or therapeutic interventions to mitigate risk and act as a protective factor against manic episodes [46, 47].

The model also suggests that decreasing an individual’s sensitivity to seasonal changes could reduce mania risk. There are several theories about how seasonality is linked to BD, including solar insolation, temperature change, and the nature of light exposure. For example, it is known that sunlight entrains human circadian systems to the natural environment and that, in individuals with a higher baseline risk of developing BD, variations in seasonal solar insolation are associated with earlier age of onset of BD-I [48]. Furthermore, it is hypothesized that modifying light exposure using blue-depleted light can reduce the risk of mania onset. Likewise, emerging evidence suggests that some individuals with BD may show abnormalities of nonvisual photoreception and that some antimanic treatments, such as lithium, may in part be effective because they decrease light hypersensitivity that is observed in some subgroups of BD patients [49, 50]. Therefore, interventions tailored to mitigate the impact of seasonal variations could be beneficial, particularly in regions with significant seasonal contrasts.

This work is not intended to be a comprehensive account of all potential mechanisms involved in the onset or recurrence of mania. Future work aims to expand the model to include other important potential causal factors, with a focus on changes in mood or cognition. This will facilitate a greater understanding of other mechanistic theories (e.g., cognitive dysfunction) and test the validity of multiple theories with existing data. The goal of the current work was to establish a foundational model for mania and bipolar disorder that can be leveraged as a collaborative research tool. This can help us determine the wider range of conditions and individual characteristics that put someone at higher risk for transition to high activation states and identify those measures that prevent or delay someone from entering the high activation states.

Our work demonstrates how we might address an important gap in understanding the causal and temporal development sequence and course of mania. While much research focuses on specific physiological or biological pathways and mechanisms, these methods can be limited in their capacity to consider complex relationships inherent to major mental disorders. The use of computational modeling here enables a complex systems approach to address these gaps and test the role of multiple interacting components in scenarios that may be hard to observe in practice due to limited prevalence, practicality, or other ethical considerations. Such a digital laboratory represents a unique synthesis between theoretical and experimental approaches in mental health, alleviating various shortfalls each of them has separately.

The utilitarian advantage of this approach over traditional statistical approaches is the explicit mechanistic operationalization of quantifiable behavioral or environmental factors relevant to mania development. This allows us to specify explicit hypotheses about discrete phenomena and the causal relationships between them. In this way, we propose a complementary perspective to traditional statistical methodologies, a language that enables us to iteratively interrogate potential mechanisms and hypotheses for the development of mania. The use of digital agents also helps to address privacy, ethical, and feasibility concerns, which can limit individualized and interventional research, as well as providing a rapid method for testing specific causal hypotheses, and the likely size of the effect of proposed interventions. In this way, an ABM represents an ideal digital laboratory that is timely, low-cost, ethical, and sustainable.

The development of computational models for mania opens new avenues for hypothesis testing and intervention simulation, offering a promising tool for advancing personalized treatment strategies. These models can facilitate the dynamic management of BD, allowing for the prediction and prevention of manic episodes through simulated scenarios tailored to individual patient profiles. This may be particularly powerful when passive sensing and Ecological Momentary Assessment (EMA) technologies are integrated with computational models to provide real-time insights about a person’s SWB and temporal patterns to quantify future risks [51]. This approach not only enhances our understanding of the causal mechanisms underlying mania but also provides a practical framework for developing targeted interventions that address the unique needs of youth with BD.

Critical next steps for validation and utilization of the model include refining the digital population using longitudinal cohort data with repeated measures of sleep-wake patterns, activity, mood, and clinical outcomes, and integrating multimodal datasets (e.g., actigraphy, self-report, and physiological measures) to better constrain individual-level dynamics. Such data will enable greater external validation of model outputs and facilitate the development of personalized digital twins that capture inter-individual variability in behavioral rhythms and risk trajectories. In parallel, the model can be extended to explore a broader range of heterogeneous and counterfactual scenarios, including alternative environmental influences, behavioral feedback structures, and intervention strategies, thereby supporting systematic hypothesis testing and translation to clinically meaningful questions.

### Limitations

Our model is constrained to focus on key observable behaviors related to rest-activity rhythms (such as sleep, activity, and rest) and does not include all factors that may be relevant for the development of mania in youth (e.g., quality of sleep, life events, timing of activities, subjective energy, mood, cognition, family history, as well as interactions between them). This includes explicit tests for any chronic or trait-like sleep–wake characteristics that may influence the timing and changes in SWB observed here. Furthermore, the data sources that inform the model parameterization come from heterogeneous population cohorts, including the adult ones. While inclusion of these factors is important, even such a limited model can be employed to demonstrate and explain key elements of mania development. These limitations exist due to the lack of available data that systematically report on causal (temporal) relationships between the mentioned (often self-reported) factors for an actual population cohort. Such relationships may vary between cohorts, and cross-cohort validation will be needed to identify generic and specific components. A further limitation of the present model is that environmental effects were restricted to light-dark entrainment of sleep timing, which may not account for the broader seasonal influences on behavior, such as changes in daily activity levels or social rhythms, which may be an important direction for future extensions. Exploring and integrating these components is the focus of future work.

## Conclusion

While major gaps exist in quantifying the mechanisms and evolution of mental disorders, we have demonstrated the utility of a computational model based on the activation hypothesis to examine putative causal processes involved in the onset and course of mania. Our longitudinal simulations indicate that of the three SWB components examined (behavioral causality, light sensitivity-seasonality, and behavioral irregularity), no mania is possible without daily irregularities in SWB. Behavioral causality may be responsible for half of the observed manic episodes. If valid, this would imply that targeted interventions to SWB, particularly those focused on reducing irregularities in activity, may significantly reduce the risk of mania.

## Supporting information

10.1192/j.eurpsy.2026.10180.sm001 Glavatskiy et al. supplementary material Glavatskiy et al. supplementary material

## Data Availability

The data produced in this study can be simulated by the developed software, which may be available online (details are pending).

## Supplementary material

The supplementary material for this article can be found at http://doi.org/10.1192/j.eurpsy.2026.10180.
