# Supplementary material for: The effect of sleep–wake behaviors on the onset of mania in youth: A computational model
Source: Eur Psychiatry. 2026 Mar 9;69(1):e49. doi: 10.1192/j.eurpsy.2026.10180 (PMC13122514; doi:10.1192/j.eurpsy.2026.10180)

**Supplementary materials**

**Model overview**

Each person in the model is represented by a digital agent. The model simulates SWB of each agent, mimicking daily actions of an actual person. The result of the simulation is a temporal profile of the agent’s activity, which approximates the coarse-grained actigraphy data for an actual person. We will refer to this temporal profile as the agent’s simulated actigraphy.

The simulation progresses in time in discrete steps, with the agents’ state being updated every step. The step size is one hour, and the duration of the simulation is one year.

The agents in the model are heterogeneous (**Figure S1**): each agent has a unique set of values of individual characteristics (see below). These characteristics affect the agent’s instantaneous state and SWB according to the set of rules and environment that are the same for all agents (see below). The resulting simulated actigraphy is unique for each agent.


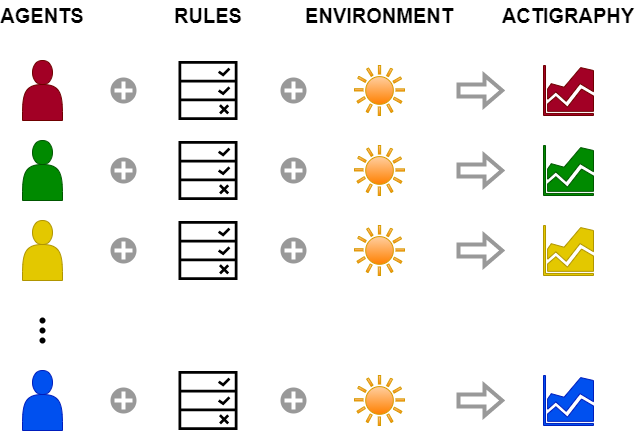


***Figure S1****: Overview of the model scope. Heterogeneous agents (indicated by different colours) follow the same behavioral rules (indicated by same rules symbol) and are being exposed to the same environment (indicated by the same sun symbol) resulting in different behaviors manifested by different simulated actigraphy (indicated by different colour).*

***Table S1:*** *SWB variables*

| **Symbol** | **Name** | **Description** |
| --- | --- | --- |
|  |  |  |
| 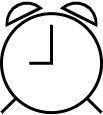 | Sleep offset | Moment at which the agent wakes up |
| 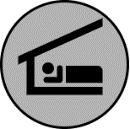 | Sleep duration | Duration of sleep |
| 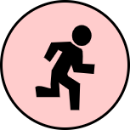 | Activity duration | Duration of all MVPA periods in a day |
| 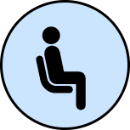 | Sedentary duration | Duration of all sedentary periods in a day |

**Agent characteristics and initialization**

Each agent possesses two types of characteristics (parameters): *cohort* and *behavioral*. The values of these parameters are non-unique and frame the bounds of agents’ SWB. Yet, each individual agent’s SWB is unique, which is ensured by the corresponding SWB variables.

The *cohort* parameters distribute the agents into categories that are typically used in population specification (**Table S2**). In addition to sex and chronotype, we introduce a binary parameter *Health*, which describes a predisposition risk to mental health issues. We do not specify the type of such risk or issues, nor intend to accurately describe it in the model – this parameter amalgamates various potential risk factors attained in the past (such as PRS, family history, childhood traumatic exposure, etc.) that may affect individual SWB.

***Table S2****: Cohort parameters*

| **Parameter** | **Description** |
| --- | --- |
| Sex | Sex: males (1) and females (0) |
| Chrono | Observed chronotype: morning (1) and evening (0) |
| Health | Predisposition risk to mental health issues: yes (1) and no (0) |

The *behavioral* parameters inform the values of the SWB variables. Each variable has three associated parameters (**Table S3**), which are the same for all agents: the default value (the population average, given all cohort parameters are the same), the heterogeneity (the standard deviation of the distribution, from which the average value of the variable for individual agent is sampled), the variability (the standard deviation of the distribution, from which the instantaneous value of the variable is sampled). Using these parameters, we model the values of the SWB variables that are unique for each agent, as described below.

***Table S3:*** *SWB parameters. Each variable has three associated parameters: Default, Heterogeneity, Variability.*

| **Associated variable** | ***Default*** | ***Heterogeneity*, h** | ***Variability*, v** |
| --- | --- | --- | --- |
| Sleep offset | 06:00 | 1.0 | 0.5 |
| Sleep duration | 8 h | 1.0 | 0.5 |
| Activity duration | 2 h | 0.4 | 1.0 |
| Sedentary duration | 10 h | 1.0 | 2.0 |

**SWB variables**

The value of each SWB variable (sleep offset, sleep duration, activity duration, sedentary duration) is calculated each day for each agent according to Equation (1), with each of the four terms corresponding to a particular contribution. Here, argument *i* refers to an individual agent and indicates that the corresponding value is unique for each agent, while argument *t* indicates the dependence on the day.

| *Value* (*i,t*) = *Reference* (*i*) + *Feedback* (*i,t*) + *Stochasticity* (*i,t*) + *Environment* (*i,t*) | (1) |
| --- | --- |

a) The *Reference* term reflects the agent’s individual reference value of the corresponding variable. It is unique for each agent and fixed for their lifetime. It represents their “typical” behavior (e.g., an agent typically wakes up at 6:35 am, or has 1h 15 min of MVPA in a day). Its value is sampled from the normal distribution with the mean and standard deviation given by Equation (2).

| *Reference* (*i*) *=* $\mathcal{N}$(*Default + Cohort, Heterogeneity*) | (2) |
| --- | --- |

Here *Default* and *Heterogeneity* are the corresponding values from **Table S3** (the same for all agents), while *Cohort* represents the correction of the population value due to agent belonging to a particular cohort, which is listed in **Table S4.**

***Table S4:*** *Cohort correction of each SWB variable. Here Sex, Chrono, Health are the parameters from* ***Table S2****, while Month is the current month in the year (1-12)*

| **Name** | ***Cohort*, h** |
| --- | --- |
| Sleep offset | + 2**Chrono – 1*Health* |
| Sleep duration | + cos(*Month*) |
| Activity duration | - 1/6*Sex |
| Sedentary duration | + 1/6*Sex |

b) The *Feedback* term reflect temporal associations between SWB variables, both within- and between- day. It represents additional change in the value of a behavioral variable on one day due to the corresponding change on the previous day. For example, if an agent has slept extra 30 minutes today, its MVPA during the day may be 5 minutes less. Let $V_{v}\left( i,t \right)$be the value of behavioral variable *v* (where *v* can be either of the four behavioral variables) for agent *i* on day *t*. Then the feedback term for each variable is calculated according to Equation (3).

| ${Feedback}_{v}(i,t) =\sum_{u} F_{vu}\cdot{[V}_{u}\left( i,t-1 \right)-V_{u}\left( i,t-2 \right)]$ | (3) |
| --- | --- |

Here $F_{vu}$ is the matrix showing the strengths of the feedback of each behavioral variable on each other behavioral variables. Its values are the same for all agents and are given in **Table S5**. The feedback matrix is also visualized in **Figure S3**.

***Table S5:*** *Feedback strength matrix. The value in each cell shows the number of minutes by which the variable in the left column is increased, if the variable in the top row is increased by 1 hour on the previous day.*

| **Variable** | Sleep offset | Sleep duration | Activity duration | Sedentary duration |
| --- | --- | --- | --- | --- |
| Sleep offset | 0 | 0 | 0 | +7 |
| Sleep duration | 0 | 0 | +18 | -11 |
| Activity duration | -4 | -2 | 0 | 0 |
| Sedentary duration | -2 | +7 | 0 | 0 |


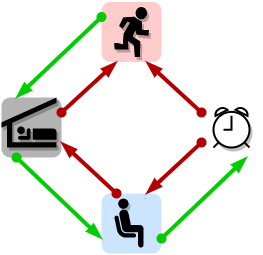


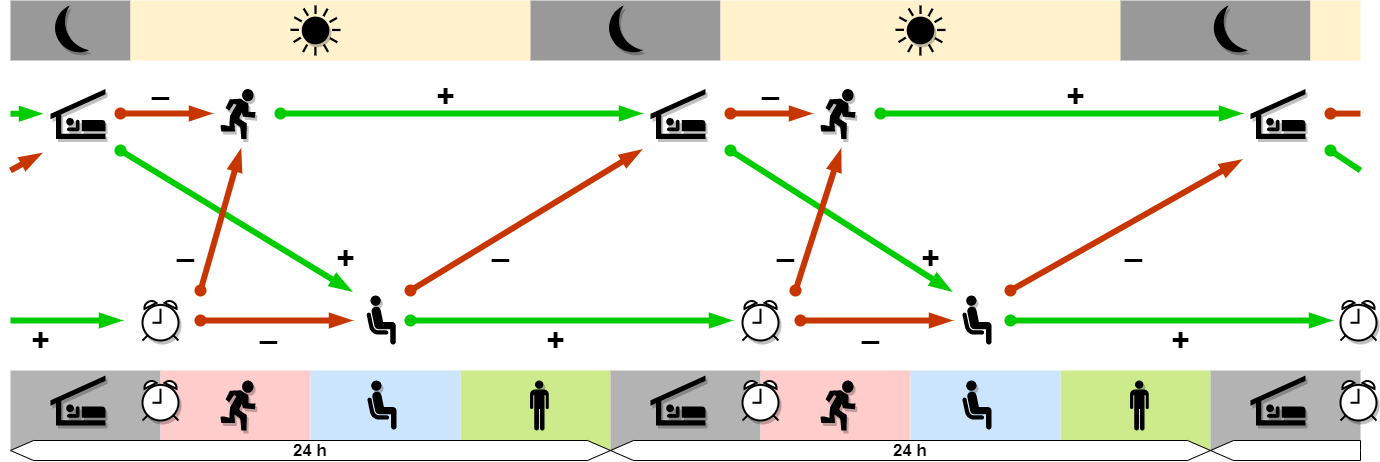


***Figure S3****: Feedback relationships between behavioral variables. Top: summary of feedback relationships, Bottom: timeline of feedback. Arrows indicate the causality (direction), sign (color: green = positive, red = negative).*

c) The *Stochasticity* term reflects day-to-day variability of the agent’s behavior. It represents a random contribution to the corresponding variable due to factors not explicitly accounted by this model. For example, while the agent’s typical wake up time is 6:35 am, the actual wake up time in a series of consecutive days may be 6:40 am, 6:41 am, 6:30 am, 6:27 am, 6:44 am due to an important meeting on the day or late party on the day before. The actual value of this term is different for each agent on every day and is sampled from the normal distribution, according to by Equation (4), with zero mean and the standard deviation given by **Table 3**.

| *Stochasticity* (*i,t*) *=* $\mathcal{N}$(*0, Variability*) | (4) |
| --- | --- |

d) The *Environment* term reflects the sunlight cycle which follows the day-night cycle and is represented by sunrise time *(ST)*, which varies throughout the year according to the geography. It is nonzero for the sleep offset variable (*SO*) only. The *Environment* term is proportional to the agent’s parameter *Sensitivity*, which characterizes the strengths of their sensitivity to the light cycle. The value of this parameter is unique for each agent and fixed for their lifetime. It is sampled from the uniform distribution, taking any value between 0 and 1. If *Sensitivity* = 0, the agent wakes up according to their individual schedule, while if *Sensitivity* = 1, the agent wakes up precisely at sunrise. In general, the environment contribution to the sleep offset variable on each day is calculated according to Equation (5).

| *Environment_SO_* (*i,t*) *= Sensitivity* (*i*) $\cdot$ [*ST* (*t*) *– SO* (*i,t*)] | (5) |
| --- | --- |

We do not distinguish weekdays and weekends or other public holidays that may disrupt the regular sleep-wake cycle of an agent. We assume such disruptions are accommodated by the *Stochasticity* term and the corresponding day-to-day variability.

**Implementation overview**

The model is implemented in the Anylogic 8.5 environment, with agents’ SWB being encoded in Java explicitly according to the details above.

The model state charts are shown in **Figure S4**. The model environment goes through day-night cycle every day, that modulates agents’ behavior. During the day-night cycle, each agent goes through the sleep-wake cycle with the changes between states happening at times determined according to the algorithm above.


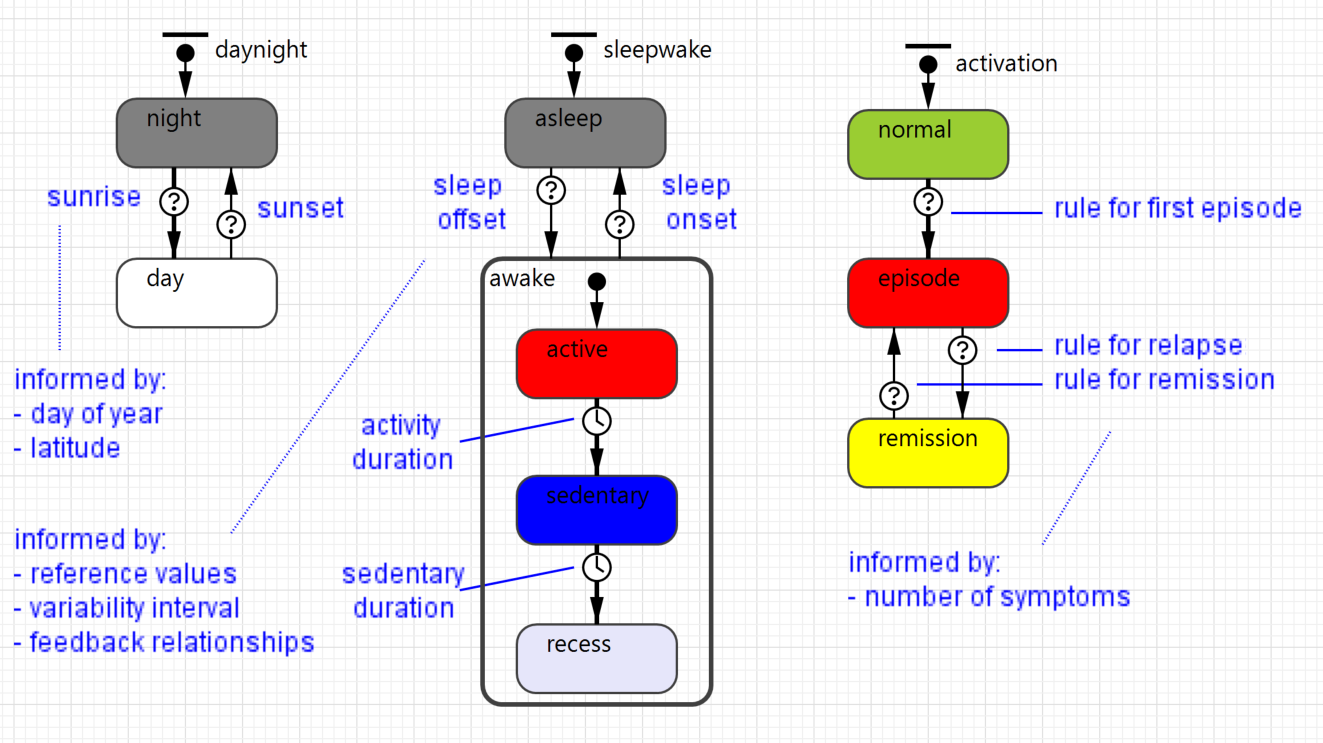


***Figure S4****: State charts of an agent: sleep-wake (responsible for individual SWB) and day-night (responsible for common day-night cycle).*

**Model interface**

The model interface, shown in **Figure S5**, consists of two sections: the control dashboard (top panel), that allows the operator changing various parameters that control individual behavior, and the output (bottom panel). The control dashboard allows changing the values of the parameters in real time, while seeing its effect instantaneously in the output panel (for a sample of 30 agents).


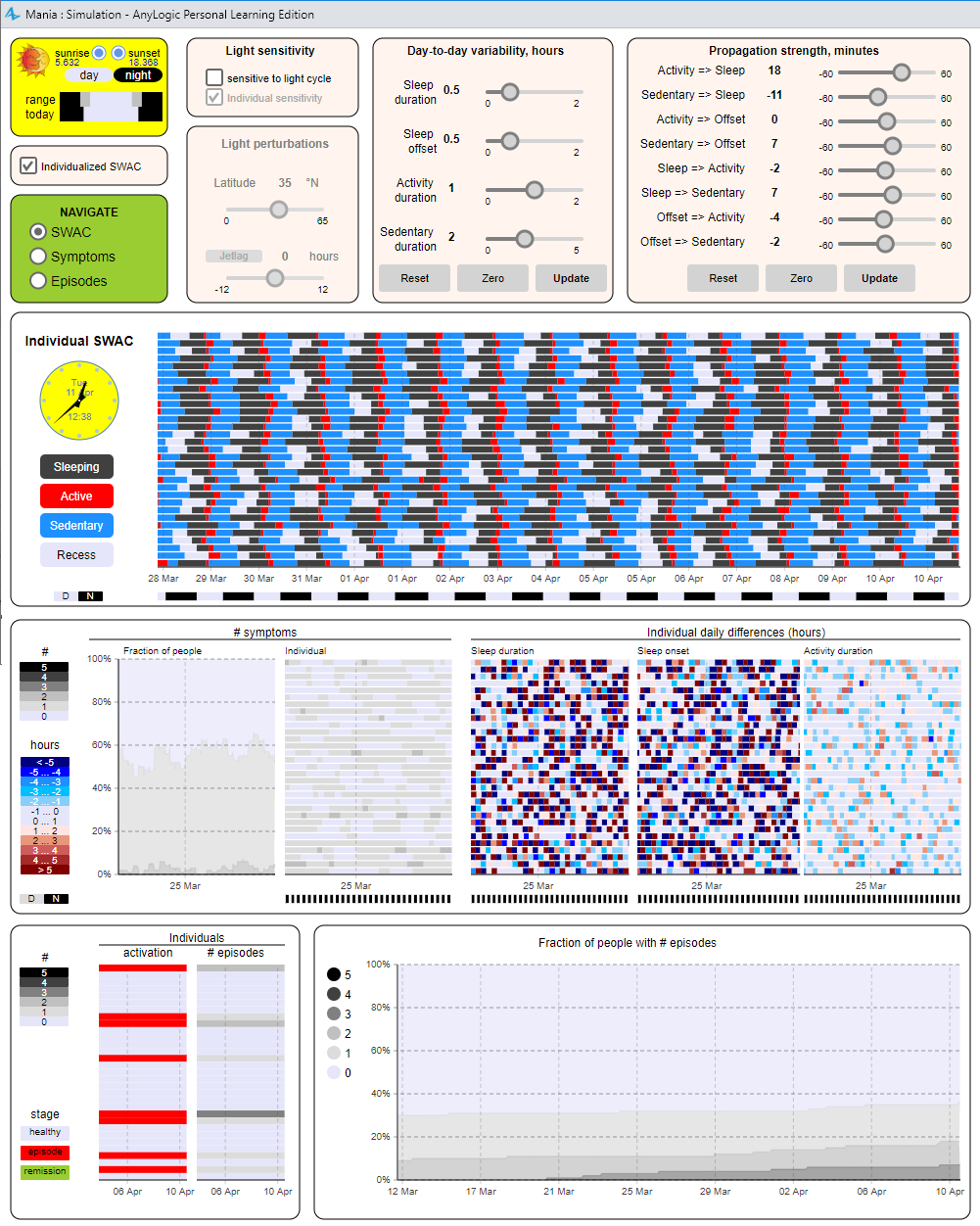


***Figure S5****: Model interface. Top panel: the control dashboard that allows the operator to change the parameters in real time. Second panel: observed SWB for a sample of 30 individuals. Each horizontal line indicates a single agent, going through four states according to Figure 2.*

The *light sensitivity* group controls individual sensitivity to day-night cycle. It includes perturbations due to travelling East-West (jetlag, implemented as an instantaneous shift in day-night cycle) and North-South (latitude, which affects the sunrise time and its variation throughout the year). The *day-to-day variability* group controls the values listed in the column “Variability” of **Table S3**. The value 0 means that an agent has no *Stochasticity* term in the corresponding variable. The *feedback strength* group controls the values listed in **Table S5**. For each pair of variables listed in the panel, it determines how many minutes the second variable changes on any day if the first variable is changed by 1 hour on the previous day. The value 0 of the strength for any pair of variables indicates that there is no relationship between these variables, while a positive (negative) value indicates a corresponding positive (negative) relationship.

**Scenario operationalization**

We identify the baseline scenario, in which the model is configured up according to the description above. In addition, we consider three complementary scenarios, in which we “remove” the corresponding terms from Equation (1): no feedback (F), no environment (E), no stochasticity (S), – as well as their combination, FE, FS, ES, FES.

In the “no feedback” scenario, we set all the coefficients from **Table S5** to zero. This essentially removes the causal links between the behavioral variables (which are illustrated in **Figure S3**) and decouples the SWB variables from each other, so that agent’s behavior on every new day is independent of their behavior on the previous day.

In the “no environment” scenario, we set *Sensitivity* parameter for all agents to zero. This essentially decouples the agent’s sleep-wake cycle from the environment (sunrise time) and its seasonal and geographical variations.

In the “no stochasticity” scenario, we set *Stochasticity* term in Equation (1) for each variable to zero. This does not prevent the variables to change (because the other terms in Equation (1) remain non-zero), but such change is becoming deterministic (still unique for each agent) rather than stochastic.

For each of the scenarios, we analyze SWB of the “average” agent, which consists of the corresponding behavioral variables averaged across all agents. We then compare the results between different scenarios. For this we calculate the average and the standard deviation (SD) of each behavioral variable over the entire simulation (one year) for the “average” agent. We refer to them as aggregate values. We then calculate the differences between these values with respect to the baseline scenario, to see the contribution of each scenario. The results are shown in **Table S6.** These results can be summarized as following.

***Table 6:*** *Differences (in minutes) between the aggregate values of SWB variables for the scenario of interest and the baseline scenario.*

| Scenario | Sleep Onset | Sleep Offset | Sleep Duration | Activity Duration | Sedentary Duration |
| --- | --- | --- | --- | --- | --- |
| F | 12.4 | 0.5 | 0.0 | 0.7 | 3.6 |
| E | 42.0 | 47.2 | 10.5 | -0.9 | -0.2 |
| E+F | 57.3 | 47.3 | 0.3 | 0.0 | 2.2 |
| S | 15.0 | 1.3 | -0.3 | 0.8 | 31.4 |
| F+S | 15.0 | 1.3 | -0.3 | 0.8 | 31.4 |
| E+S | 63.2 | 49.5 | -0.4 | 0.8 | 31.4 |
| F+E+S | 63.3 | 49.4 | -0.4 | 0.8 | 31.4 |

1) There is essentially no variation in *activity duration* across different scenarios, including the baseline one. This means that neither the behavioral feedback, nor environment, or stochasticity influences the activity duration. This is surprising, because the behavioral feedback and stochasticity explicitly affect the daily activity duration for each individual agent. Still, this is expected for the environment sensitivity, as it does not explicitly affect the individual activity duration.

2) A substantial increase (31.4 min) of *sedentary duration* compared to the baseline scenario is observed in all scenarios with no stochasticity of the behavioral variables. The stochasticity component appears to dominate over the other components of behavioral variables that contribute to Equation (1). Yet, *sedentary duration* appears to be insensitive to the behavioral feedback, which is also surprising.

3) Some increase (10.5 min) of *sleep duration* compared to the baseline scenario is observed only in the scenario with no environmental sensitivity alone (not in combination with other scenarios). Furthermore, only in this scenario SD of sleep duration (see Appendix) is increased with respect to the baseline scenario (from 24.8 min to 36.4). This indicates that the environment (in the form of sunrise time alone) has a stabilizing effect on sleep duration.

4) Substantial increase (47-49 min) of *sleep offset* compared to the baseline scenario is observed in all scenarios with no environmental sensitivity. This also applies to combined scenarios in which environmental sensitivity is only one of the absent components.

5) Substantial increase of *sleep onset* compared to the baseline scenario is observed in all alternative scenarios. This may be attributed to the fact, that in the simulation we do not control, but calculate the sleep onset.

When analyzing the individual scenarios alone, it is worth noting that having no stochasticity significantly reduces the yearly SD of behavioral variables, with some variation being present due to daylight changing transitions and variation of the sunrise time. Having no stochasticity in combination with no environmental sensitivity reduces these SD essentially to zero, as expected.

**Figure S6** shows typical trajectories of relevant variables that are used in the analysis, from a random agent. We can see that both the duration and the timing of each state varies from day to day, fluctuating around some reference value. Because the reference values for each variable and the fluctuating pattern are unique for each agent, the resulting trajectory is also unique for each agent. *Top: the timing of sleep onset and offset. Bottom: the duration of asleep, active, and sedentary states.*


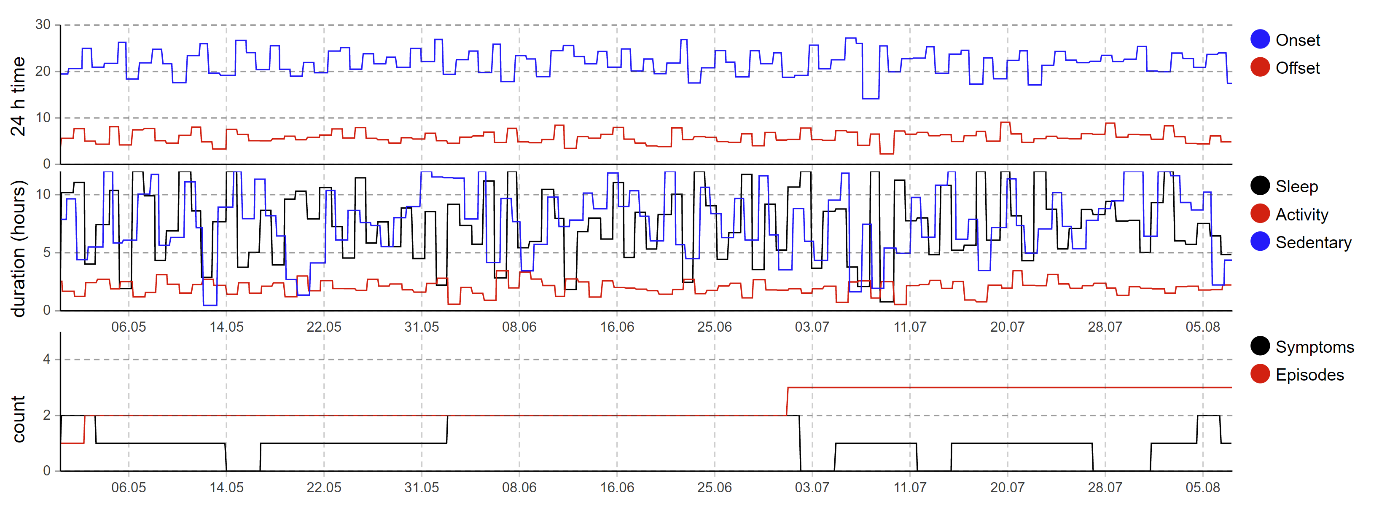


**Figure S7** shows the same kind of output as **Figure S6**, but for the average agent and over the course of the entire simulation. The value of each variable fluctuates around the corresponding default value. The average value for the duration variables is stable across the year, while the sleep onset and offset shift by 1 hour at the daylight savings transitions. The magnitude of the fluctuation is different for each variable, with the largest being for the durations of asleep and sedentary states. This is determined by 1) the corresponding values of *Heterogeneity* and *Variability* columns from **Table 3**, and 2) the magnitude of the coefficients from **Table 5**.

In addition, we can notice that magnitude of the sleep onset fluctuations is larger than the one of sleep offset. This is because the former is not controlled through Equation (1), but calculated to balance 24h-day, hence being affected by the fluctuations in all four variables together. *Top: the duration of asleep, active, sedentary states. Bottom: the timing of sleep onset and offset.*


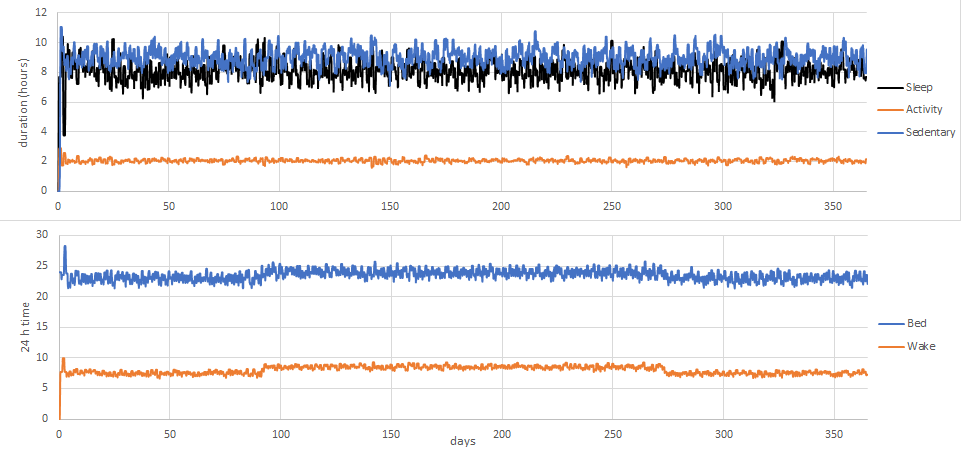

Supplement: Glavatskiy et al. supplementary material [file S0924933826101801sup001.docx]
